# POWERFUL TOOLS FOR CAREGIVERS: THE ESTABLISHED BENEFITS OF A PERSON-CENTERED CARE INTERVENTION

**DOI:** 10.1093/geroni/igad104.1227

**Published:** 2023-12-21

**Authors:** Naomi Meinertz

**Affiliations:** University of Missouri, Columbia, Missouri, United States

## Abstract

Family caregivers account for 89% of caregivers in the United States, yet few evidence-based family care programs focus specifically on the caregiver’s needs rather than the care receiver. The challenges that caregivers experience, such as increased depression symptoms and feelings of burden, impede their ability to fulfill their role as care partner and can lead to lasting health and financial issues. Intervention literature underscores the importance of using person-centered models to incite behavior change, yet many caregiver interventions do not address the caregiver’s needs and instead approach caregiving from a practical perspective to caregiving such as establishing a power of attorney or educating caregivers about dementia. Powerful Tools for Caregivers (PTC) is a six-week program designed to provide caregivers with the skills to address their unique care challenges. Within in-person or virtual sessions, caregivers are provided with the skills to improve caregiver self-efficacy, emotional management, self-care, and resource access and knowledge. The study integrates empirical findings and demonstrates the effectiveness of PTC in improving caregiver well-being in each focus area. Findings also highlight the benefits of the group-based activities as well as improved depressive symptoms and reduced caregiver stress and burden. This research also demonstrates the same effectiveness of delivery format (in-person or virtual mediums) and location (rural or urban settings). The success of PTC sheds light on the need for more programs to approach family care through the lens of a person-centered model and emphasizes the benefit of evidence-based programs.

